# Intraoperative Correlates of Electrode Array Insertion Dynamics and Electrophysiological Parameters Under SmartNav-Guided Cochlear Implantation: An Exploratory Single-Centre Study

**DOI:** 10.3390/diagnostics16213464

**Published:** 2026-10-23

**Authors:** Cristian Mircea Neagoș, Eugenia Maria Domuța, Adriana Neagoș, Ileana Anca Sin

**Affiliations:** 1 Doctoral School of Medicine and Pharmacy, George Emil Palade University of Medicine, Pharmacy, Science, and Technology of Targu Mures, 38 Gheorghe Marinescu Street, 540142 Targu Mures, Romania; neagos.cristian-mircea.23@stud.umfst.ro; 2 Department of Surgery, Faculty of Medicine and Pharmacy, University of Oradea, 10 1 Decembrie Square, 410073 Oradea, Romania; maria.domuta@yahoo.com; 3 Department of Otorhinolaryngology, George Emil Palade University of Medicine, Pharmacy, Science, and Technology of Targu Mures, 38 Gheorghe Marinescu Street, 540142 Targu Mures, Romania; 4 Department of Molecular Biology, George Emil Palade University of Medicine, Pharmacy, Science, and Technology of Targu Mures, 38 Gheorghe Marinescu Street, 540142 Targu Mures, Romania; ileana.sin@umfst.ro

**Keywords:** cochlear implantation, electric impedance, electrodes, implanted, evoked potentials, auditory, neuromonitoring

## Abstract

**Background/Objectives:** Real-time intraoperative navigation systems such as SmartNav (Cochlear Ltd., Sydney, Australia) provide objective feedback on electrode array insertion during cochlear implantation, yet it remains unclear whether the parameters they record vary across the device- and patient-level factors a surgeon selects between. This study compared intraoperative insertion dynamics and electrophysiological parameters across electrode array design (CI622 slim-straight versus CI632 slim-modiolar), surgical approach (round window versus cochleostomy), and patient age (children versus adults). **Methods:** Twenty-nine consecutive implantations in 26 patients (three children implanted bilaterally) performed with SmartNav monitoring were analysed. Insertion angle, insertion speed, total insertion time, the number of contacts (of 22) with in-range impedance, and the number of contacts (of 22) with a measurable neural response telemetry (NRT) response were extracted from device logs and operative records; insertion kinetics were available for 22 of 29 implantations. Distributions were non-normal, so comparisons used the Mann–Whitney U test with rank-biserial effect sizes, Fisher’s exact test, Kruskal–Wallis test, and Spearman correlation. Exploratory multivariable regression, Brown–Forsythe variance testing, bootstrap effect sizes, and outlier-inclusive and one-ear-per-patient sensitivity analyses were additionally applied; no correction for multiple comparisons was made. **Results:** The CI632 array was advanced faster per electrode than the CI622 (median 0.33 versus 0.56 s/electrode; *n* = 5 versus 16; *p* = 0.015; rank-biserial r = −0.75; bootstrap 95% CI −1.00 to −0.38), but the difference was not significant when one implausible outlying value was retained (*p* = 0.113). Impedance and NRT status were modestly correlated (ρ = 0.388, *p* = 0.037, uncorrected) and unchanged after adjustment for age band (partial ρ = 0.389), but were not significant in the one-ear-per-patient analysis. The study was unable to detect a difference between age groups or between surgical approaches (five cochleostomies versus 24 round window insertions) in any parameter, and impedance status varied minimally across all strata. **Conclusions:** Within the limits of a small, unbalanced cohort, SmartNav-derived parameters did not differ detectably across patient age or surgical approach. The data suggest that electrode array design is associated with per-electrode insertion pacing, an observation that depends on a single excluded data point and requires confirmation. The impedance–NRT association is exploratory, is consistent with a chance finding once multiplicity is considered, and should not yet be regarded as an intraoperative integrity signal. No clinical outcome was linked to the intraoperative parameters, so no inference about the clinical superiority of either array or approach can be drawn.

## 1. Introduction

Sensorineural hearing loss of cochlear origin is among the most prevalent sensory impairments worldwide and, when severe to profound, cannot be adequately rehabilitated with conventional acoustic amplification [1]. Cochlear implantation has therefore become the established standard of care for this population, directly stimulating the auditory nerve and restoring functional hearing in both children and adults, a position now formalised in international consensus statements [2]. Systematic evidence confirms durable gains in speech perception, communication, and health-related quality of life following implantation [3].

As implantation indications have broadened and hearing preservation has become an explicit surgical goal, the emphasis of intraoperative practice has shifted from simply achieving full electrode insertion toward achieving an atraumatic, controlled, and reproducible insertion that protects residual cochlear structures. The feasibility of this depends closely on cochlear morphology, which varies considerably between individuals in duct length, curvature, and scalar cross-section [4]. Histologically controlled studies established early that the route and manner of entry into the scala tympani materially influence the degree of intracochlear trauma sustained during array placement [5].

Atraumatic insertion depends on a small number of measurable mechanical quantities: the angular depth achieved, the speed at which the array is advanced, and the total time spent within the scala. Experimental work has demonstrated that insertion forces rise steeply with advancement velocity [6], and that faster insertion is associated with poorer preservation of inner-ear function [7]. Intracochlear pressure transients generated during array advancement provide a further mechanistic link between insertion kinetics and cochlear injury [8]. Historically, these quantities were inferred indirectly or judged subjectively by the operating surgeon.

The introduction of intraoperative navigation and telemetry platforms has made it possible to capture these quantities objectively and in real time. The SmartNav system integrates insertion kinetics measurement with impedance telemetry and evoked neural response telemetry (NRT) within a single perioperative workflow, providing the surgeon with immediate numerical feedback on insertion speed, positioning, and electrode function [9]. Reported advantages include improved surgical efficiency and reduced dependence on a dedicated audiologist in the operating room [10]. Most clinical reports to date, however, have presented SmartNav output as pooled summary statistics without formally testing whether these parameters differ across the factors surgeons actually choose between.

Three such factors are of particular practical interest. First, electrode array design differs mechanically: the CI622 is a lateral wall slim-straight array, whereas the CI632 is a pre-curved slim-modiolar array deployed by a sheath-based controlled-release technique; array design and surgical approach jointly determine scalar location and downstream outcome [11]. Second, the surgical entry route—round window versus cochleostomy—remains the subject of an enduring debate regarding intracochlear trauma and final electrode position [12]. Third, patient age at implantation, and specifically the distinction between paediatric and adult recipients, remains central to counselling and to the rationale for early implantation.

Beyond simple group differences, the internal structure of the intraoperative dataset is itself informative. Impedance and NRT both reflect the integrity of the electrode–neural interface, and an empirical association between them would be a first step towards evaluating their combined use as a real-time integrity signal. Accordingly, the objectives of the present study were (i) to compare insertion angle, speed, time, impedance, and NRT between electrode designs, surgical approaches, and age groups using appropriate non-parametric inference; (ii) to quantify the internal correlation structure of these parameters and test its robustness to confounding; and (iii) to apply exploratory multivariable modelling, procedural consistency analysis, bootstrap effect size estimation, and sensitivity analyses in order to characterise both the magnitude and the stability of any observed differences. Given the sample size, the study was designed as hypothesis-generating, and its results are interpreted accordingly.

## 2. Materials and Methods

### 2.1. Study Design and Setting

This was an observational, comparative study conducted within the Clinical Department of Otorhinolaryngology of the Emergency County Clinical Hospital, Târgu Mureș, Romania. Consecutive patients who underwent cochlear implantation with intraoperative SmartNav monitoring between December 2023 and October 2025 were eligible for inclusion. The final analytic cohort comprised 29 implantations in 26 unique patients: 16 children and 10 adults, of whom three children received sequential bilateral implants and therefore contributed two ears each (19 paediatric and 10 adult ears; 16 male and 13 female ears). No patient contributed a repeat (revision) implantation of the same ear. All procedures were performed by the same surgical team using a standard transmastoid posterior tympanotomy approach under general anaesthesia.

Inclusion criteria were a diagnosis of severe-to-profound sensorineural hearing loss, implantation performed at the study institution, intraoperative use of the SmartNav system, and availability of the intraoperative electrophysiological data (impedance and NRT). Exclusion criteria were anatomical cochlear malformation, active inner-ear disease or complication, and absent SmartNav data. Insertion kinetics parameters (angle, speed, and time) were analysed on an available-case basis, because the kinetics record was not saved in every session (Section 2.2); baseline characteristics and the completeness of each parameter by array are tabulated in the Results (Table 1 and Table 2). The three second-ear (contralateral) records were treated as independent implantation events in the primary analysis; a one-ear-per-patient sensitivity analysis retaining only the first-implanted ear of each patient (*n* = 26) was reported in Section 3, and the implications of this decision are addressed explicitly in the limitations.

### 2.2. Variables and Data Sources

Five intraoperative quantities were analysed. The insertion angle, expressed in degrees of angular insertion depth, is computed by the SmartNav platform only for the CI622 array, as angular depth is not derived for the pre-curved CI632; angle analyses are therefore restricted to CI622 cases. Insertion speed was expressed in seconds per electrode and total insertion time in seconds; the two are computed differently by the platform. As the array is advanced, each contact registers a transition from an open-circuit reading to an intracochlear (fluid) impedance, and SmartNav time-stamps these contact-entry events. The insertion-speed value analysed here is the platform’s summary of the mean interval between successive contact-entry events during the advancement phase, so that it reflects the pace at which contacts were delivered into the cochlea while the array was actively moving; a lower value denotes faster advancement. Total insertion time is the elapsed interval between the first contact-entry event and the surgeon’s confirmation of completed insertion on the tablet, and therefore additionally includes any pauses, repositioning, and tactile reassessment and, for the CI632, the sheath-based release manoeuvre and sheath withdrawal, during which no further contact-entry events occur. The two quantities are consequently not simple transformations of one another, and an array can show a shorter per-electrode interval yet a longer total time. Impedance status was expressed as the number of intracochlear contacts (of 22) whose impedance at the intraoperative Common Ground impedance check lay within the manufacturer’s normal range, that is, contacts flagged as neither open- nor short-circuit; NRT status was expressed as the number of contacts (of 22) at which a measurable electrically evoked compound action potential (ECAP) was obtained with the automated NRT (AutoNRT) protocol at the intraoperative measurement point. Both are the array-level summary values displayed on the SmartNav intraoperative report; neither is an impedance magnitude in kΩ nor an ECAP threshold in current-level units, and a value of 22 represents the ceiling of the scale (all contacts within range, or all contacts responding) rather than a measured magnitude. Both were therefore treated as bounded ordinal counts, and the resulting heavy tying at the ceiling is taken into account in the interpretation of the correlation analyses (Section 3).

Data were drawn from three concordant sources: the intraoperative SmartNav session logs, the surgical operative notes, and the postoperative audiological records. Patient- and device-level grouping variables—sex, ear side, paediatric versus adult status, three-level age band (0–6, 6–10, and >10 years), electrode design (CI622 versus CI632), and surgical approach (round window versus cochleostomy)—were transcribed from the operative record. One insertion-speed value of 4.5 s/electrode, recorded for a CI632 implantation in an adult inserted through the round window, lay more than four standard deviations above the remainder of the distribution. The value is mechanically implausible because it is internally inconsistent with the same session log: at 4.5 s per contact, delivery of the 22 contacts would have required approximately 99 s of active advancement, whereas the total insertion time logged for that case was 38 s. A misplaced decimal point (0.45 s/electrode) would be consistent with the logged total time, but because the correct value could not be verified, it was neither imputed nor corrected; it was treated as a data-entry artefact and excluded from speed analyses, with a sensitivity analysis retaining it reported for transparency. The total insertion time and electrophysiological values from that case, which were consistent with the log, were retained.

The insertion kinetics record (angle, speed, and time) was available for 22 of the 29 implantations: 16 of 19 CI622 and 6 of 10 CI632 sessions. In the seven sessions without a kinetics record, the kinetics module had either not been armed on the tablet before array advancement began (five sessions, all within the first four months of the study period and corresponding to the team’s familiarisation with the platform) or the session export was incomplete (two sessions). In none of the seven was the absence of the record related to an intraoperative event, a difficult, incomplete, or aborted insertion, or re-deployment of the array; all seven insertions were completed uneventfully at the first attempt. The proportion of sessions with a missing kinetics record did not differ significantly between arrays (3 of 19 versus 4 of 10; Fisher’s exact *p* = 0.193), although the absolute proportion was higher for the CI632, and the resulting small CI632 kinetic subgroup (*n* = 5 for speed after outlier exclusion, *n* = 6 for time) is the principal constraint on the electrode array comparison. Impedance and NRT status, which are acquired after full insertion, were available for all 29 implantations.

### 2.3. Statistical Analysis

Continuous variables are summarised as median and interquartile range (IQR) alongside mean and standard deviation, and categorical variables as counts and percentages. The distributional shape of each continuous variable was assessed with the Shapiro–Wilk test; insertion speed, insertion time, impedance, and NRT all deviated significantly from normality (*p* < 0.05), so non-parametric methods were adopted throughout. Two-group comparisons of continuous variables used the Mann–Whitney U test. Comparisons across the three age bands used the Kruskal–Wallis test. Associations between categorical variables used Fisher’s exact test, which is preferable to the chi-square test given the small expected cell counts. Relationships between continuous parameters were quantified with Spearman’s rank correlation coefficient.

Three additional strategies were applied to characterise the magnitude and stability of observed differences; all were exploratory. A multivariable ordinary-least-squares regression modelled total insertion time as a function of age group, surgical approach, electrode design, and insertion speed, with standardised coefficients reported to permit comparison of relative influence. This model was intended solely as an exploratory adjustment analysis to examine whether the univariable patterns persisted after mutual adjustment; with 21 observations and four predictors it is markedly underpowered and was not designed to identify independent determinants of insertion time. Procedural consistency was examined with the Brown–Forsythe variant of Levene’s test alongside the coefficient of variation, on the rationale that a standardising technology should compress between-case variability. Finally, group differences were quantified using the Hodges–Lehmann median-difference estimator [13] and the rank-biserial correlation as a distribution-free effect size [14], each accompanied by 2000-sample bootstrap percentile confidence intervals. All tests were two-sided with α = 0.05; analyses used Python 3.12 with the SciPy and statsmodels libraries. In total, 51 inferential tests are reported (4 Fisher’s exact tests, 14 Mann–Whitney comparisons, 5 Kruskal–Wallis tests, 10 pairwise and 2 within-array Spearman correlations, 1 partial correlation, 5 regression tests, 8 Brown–Forsythe tests, 1 dichotomised comparison, and 1 outlier-inclusive sensitivity test). No formal correction for multiplicity was applied because the study was designed as hypothesis-generating; for reference, a Bonferroni-adjusted threshold across all 51 tests would be α ≈ 0.001, and no result reaches it. Two further sensitivity analyses were added during peer review: the electrode array comparison with the excluded speed value retained, and a one-ear-per-patient analysis in which only the first-implanted ear of each bilaterally implanted patient was retained (*n* = 26).

### 2.4. Ethical Considerations and Reproducibility

The study protocol was approved by the Ethics Committee of the Emergency County Clinical Hospital, Târgu Mureș, Romania. The study used routinely collected clinical and device-generated data, handled in de-identified form. No intervention was allocated for research purposes and no change to clinical management followed from the analysis. Data were stored on institutional systems in accordance with local data-protection requirements, and results are reported at the group level so that no individual can be identified from any table or figure.

To support reproducibility, every reported *p*-value, effect size, and confidence interval was computed directly from the primary dataset using documented, scriptable procedures, and the exact test used is named beside each result in the tables that follow. The single excluded speed value is disclosed, and its influence is quantified in a dedicated sensitivity analysis, so that both the primary and the outlier-inclusive result can be reconstructed.

## 3. Results

The analytic cohort of 29 implantations in 26 patients comprised 19 paediatric ears (65.5%) and 10 adult ears (34.5%), with 16 male (55.2%) and 13 female (44.8%) ears; three children contributed both ears. Angular insertion depth was measurable in 16 CI622 cases (mean 318.9°, SD 39.8°). Insertion speed after outlier exclusion had a median of 0.49 s/electrode (IQR 0.33–0.70; *n* = 21), total insertion time ranged from 15 to 123 s (median 30 s, SD 26.8 s; *n* = 22), impedance status had a mean of 21.69 contacts in range (SD 0.93; 22 of 22 in 25 implantations and 18, 19, 21, and 21 in the remaining four), and NRT status a mean of 20.24 responding contacts (SD 2.52; range 11–22, with 22 of 22 in 14 implantations). Baseline demographic and clinical characteristics are summarised in Table 1, and the availability of each parameter by electrode array in Table 2.

No baseline characteristic differed significantly between age groups: sex (*p* = 1.000), ear side (*p* = 0.270), electrode design (*p* = 0.698), or surgical approach (*p* = 0.306). The round window approach was used in 24 of 29 implantations (82.8%) and the CI622 array in 19 (65.5%). Thirteen recipients (44.8%) were implanted before six years of age. The age group comparisons are detailed in Table 3.

No intraoperative parameter differed significantly between children and adults. Insertion angle medians were 297.0° and 324.0° (U = 25.0, *p* = 0.821), insertion speed 0.49 and 0.43 s/electrode (*p* = 1.000), and insertion time 30.0 and 27.0 s (*p* = 0.805). Impedance status was 22 of 22 contacts in both groups (*p* = 0.646) and NRT medians were 21.0 and 22.0 responding contacts (*p* = 0.733). All rank-biserial effect sizes were 0.09 or lower. Comparisons by surgical approach are presented in Table 4.

The study was unable to detect a difference between surgical approaches in any parameter (5 cochleostomies versus 24 round window insertions). Insertion angle medians were 297.0° for round window and 324.0° for cochleostomy (*p* = 0.496) and insertion speed 0.52 and 0.37 s/electrode (*p* = 0.509). Insertion time medians were 30.0 and 27.0 s (*p* = 0.814), impedance status was 22 of 22 contacts in both groups (*p* = 0.360), and NRT medians were 21.5 and 20.0 responding contacts (*p* = 0.461). Effect sizes ranged from 0.08 to 0.24, with wide confidence intervals that include zero (see the effect size synthesis below); the comparison is underpowered, and non-significance should not be read as equivalence. Comparisons by electrode array design are presented in Table 5.

**Figure 1:**
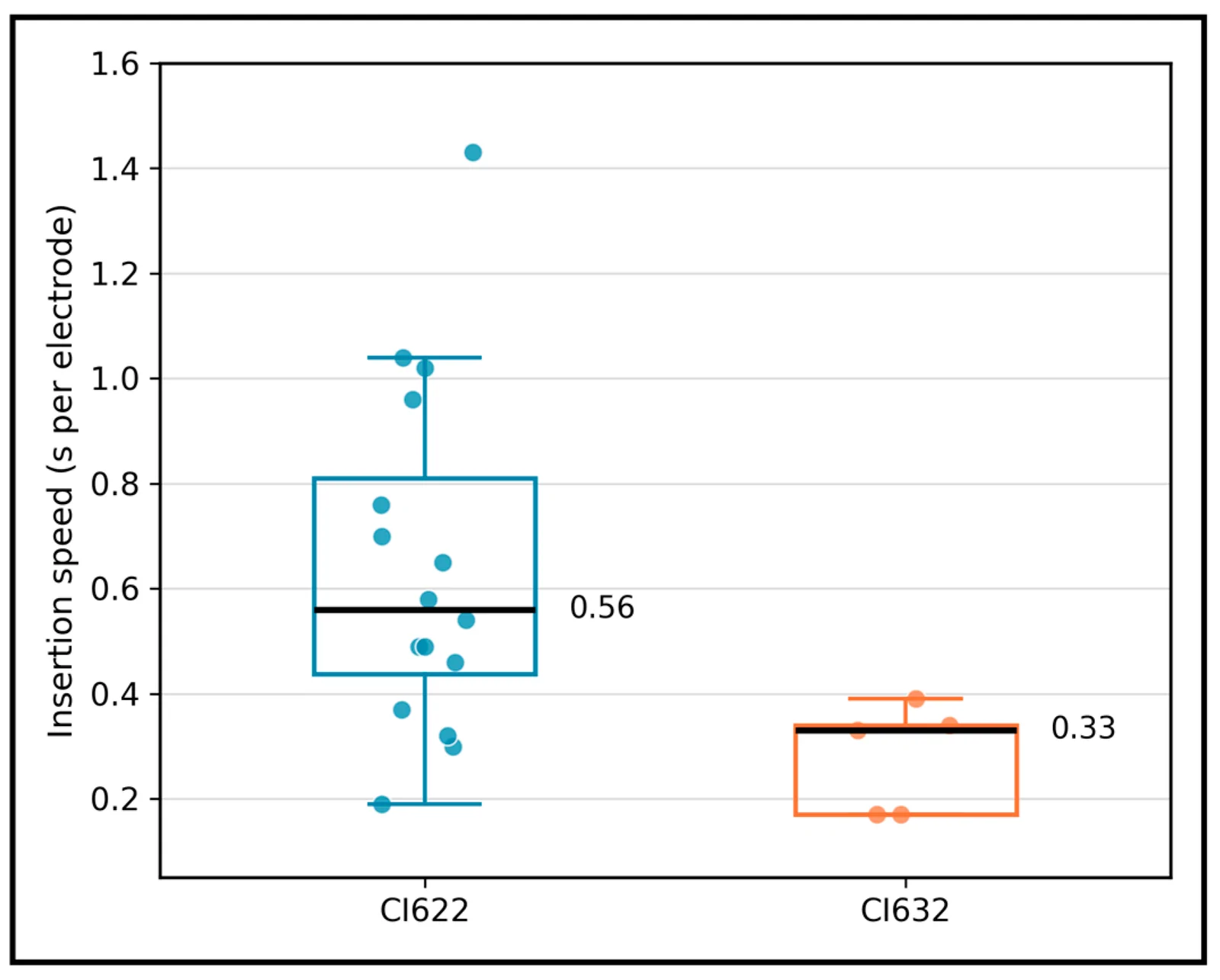
Insertion speed by electrode array. Boxes span the interquartile range with the median as a horizontal line and whiskers extending to the most extreme values within 1.5 × IQR; every implantation is plotted as an individual point. Median values are labelled. Lower values denote faster per-electrode deployment.

**Figure 2:**
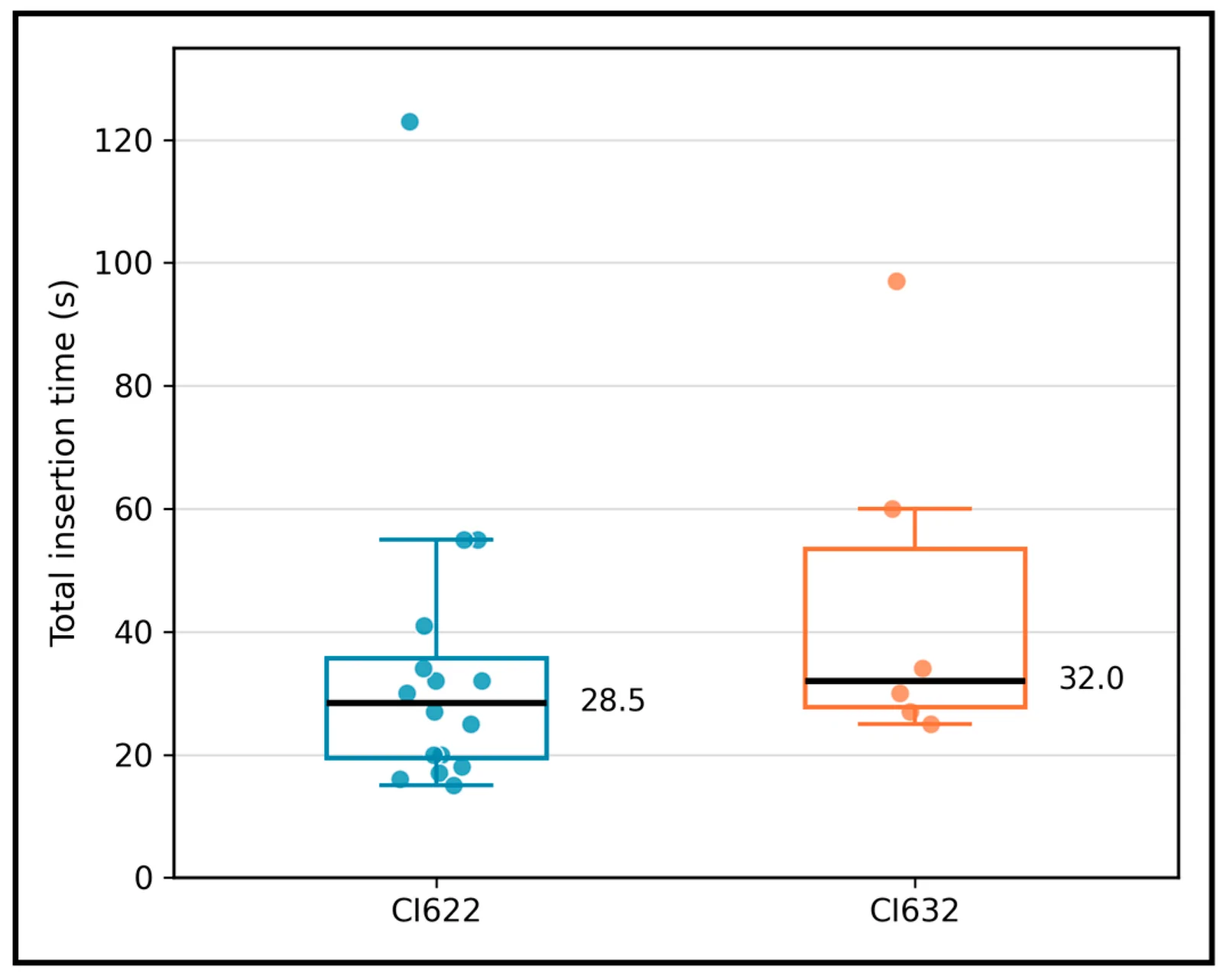
Total insertion time by electrode array. Boxes span the interquartile range with the median as a horizontal line and whiskers extending to the most extreme values within 1.5 × IQR; every implantation is plotted as an individual point. Median values are labelled.

Insertion speed differed between electrode arrays in the primary (outlier-excluded) analysis: median 0.56 s/electrode for CI622 (*n* = 16) versus 0.33 s/electrode for CI632 (*n* = 5) (U = 70.0, *p* = 0.015), with a rank-biserial effect size of −0.75; the difference was not significant when the excluded value was retained (see Exploratory and Sensitivity Analyses below). No other parameter reached significance: insertion time 28.5 versus 32.0 s (*p* = 0.252), impedance status 22 of 22 contacts in both arrays (*p* = 0.491), and NRT 21.0 versus 22.0 responding contacts (*p* = 0.294). The distributions underlying the two mechanical parameters are shown in Figure 1 and Figure 2.

Insertion speed was separated by array in the 21 implantations with an analysable value. Median values were 0.56 s/electrode for CI622 (IQR 0.44–0.81; *n* = 16) and 0.33 s/electrode for CI632 (IQR 0.17–0.34; *n* = 5). Individual CI622 values ranged from 0.19 to 1.43 s/electrode, whereas no CI632 value exceeded 0.39 s/electrode.

Total insertion time did not differ by array. Median values were 28.5 s for CI622 (IQR 19.5–35.8; *n* = 16) and 32.0 s for CI632 (IQR 27.8–53.5; *n* = 6). Both distributions were right-skewed, with maxima of 123 s and 97 s respectively. The dissociation between a shorter per-electrode interval and a longer total time for the CI632 reflects the different composition of the two metrics (Section 2.2): total time includes the sheath-release and withdrawal steps, during which no contact-entry events occur, whereas the per-electrode interval captures only the advancement phase. The full correlation matrix is provided in Table 6.

**Figure 3:**
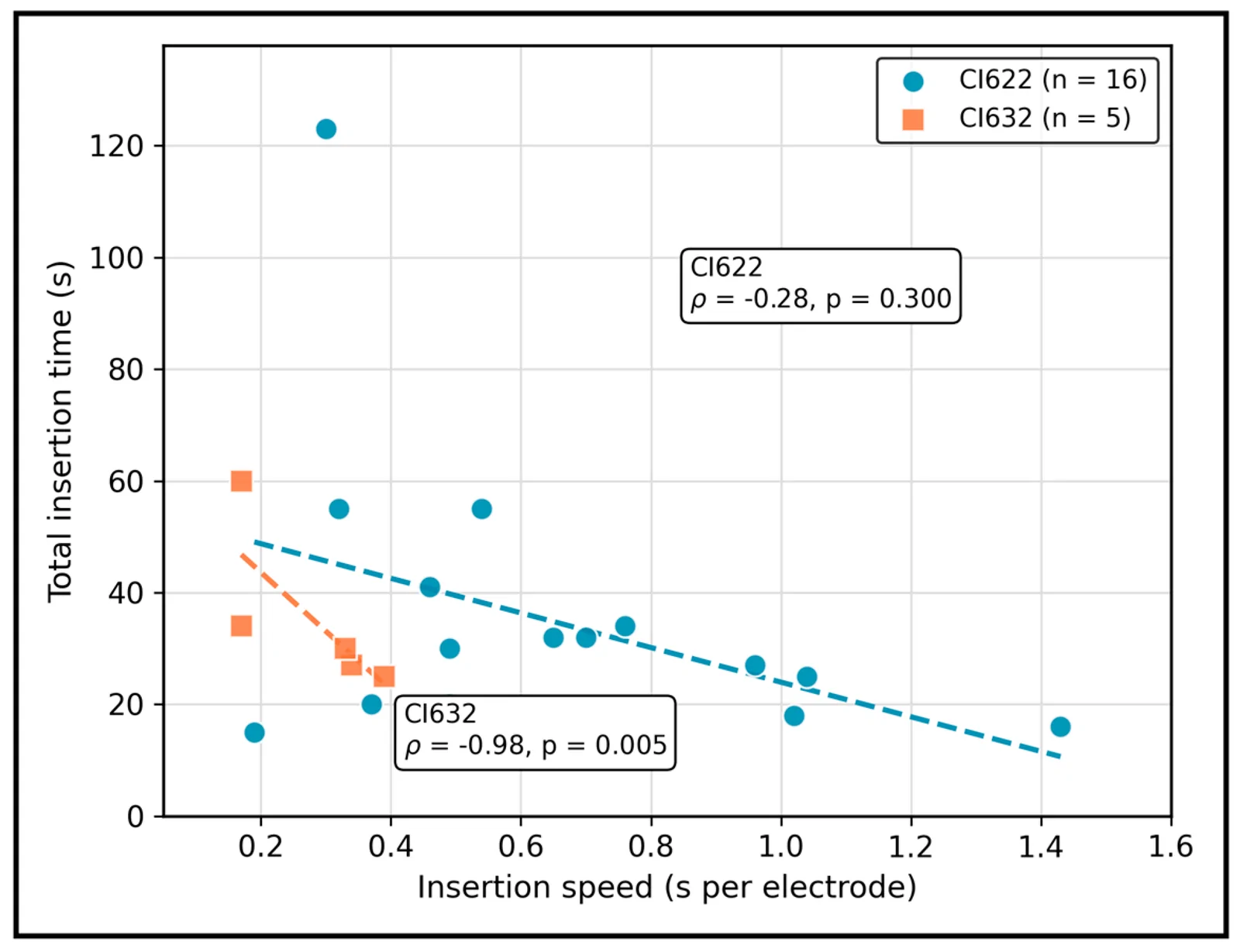
Relationship between insertion speed and total insertion time, plotted separately for each electrode array with least-squares trend lines. Spearman coefficients for each array are annotated. CI622 *n* = 16; CI632 *n* = 5. The CI632 trend line and coefficient rest on only five points and are exploratory.

One of the ten parameter pairs reached uncorrected significance: impedance and NRT status (ρ = 0.388, *p* = 0.037). Because impedance status takes the ceiling value in 25 of 29 implantations, this coefficient is effectively a comparison of the four sub-ceiling cases with the remainder and is equivalent to the dichotomised comparison reported below; with 51 tests reported in this study, a single association at *p* = 0.037 is consistent with a chance finding. Insertion speed and total insertion time were inversely related without reaching significance (ρ = −0.381, *p* = 0.089). Insertion angle correlated negatively with impedance (ρ = −0.365, *p* = 0.165) and with NRT (ρ = −0.370, *p* = 0.159). The speed–time relationship is shown separately by array in Figure 3.

**Figure 4:**
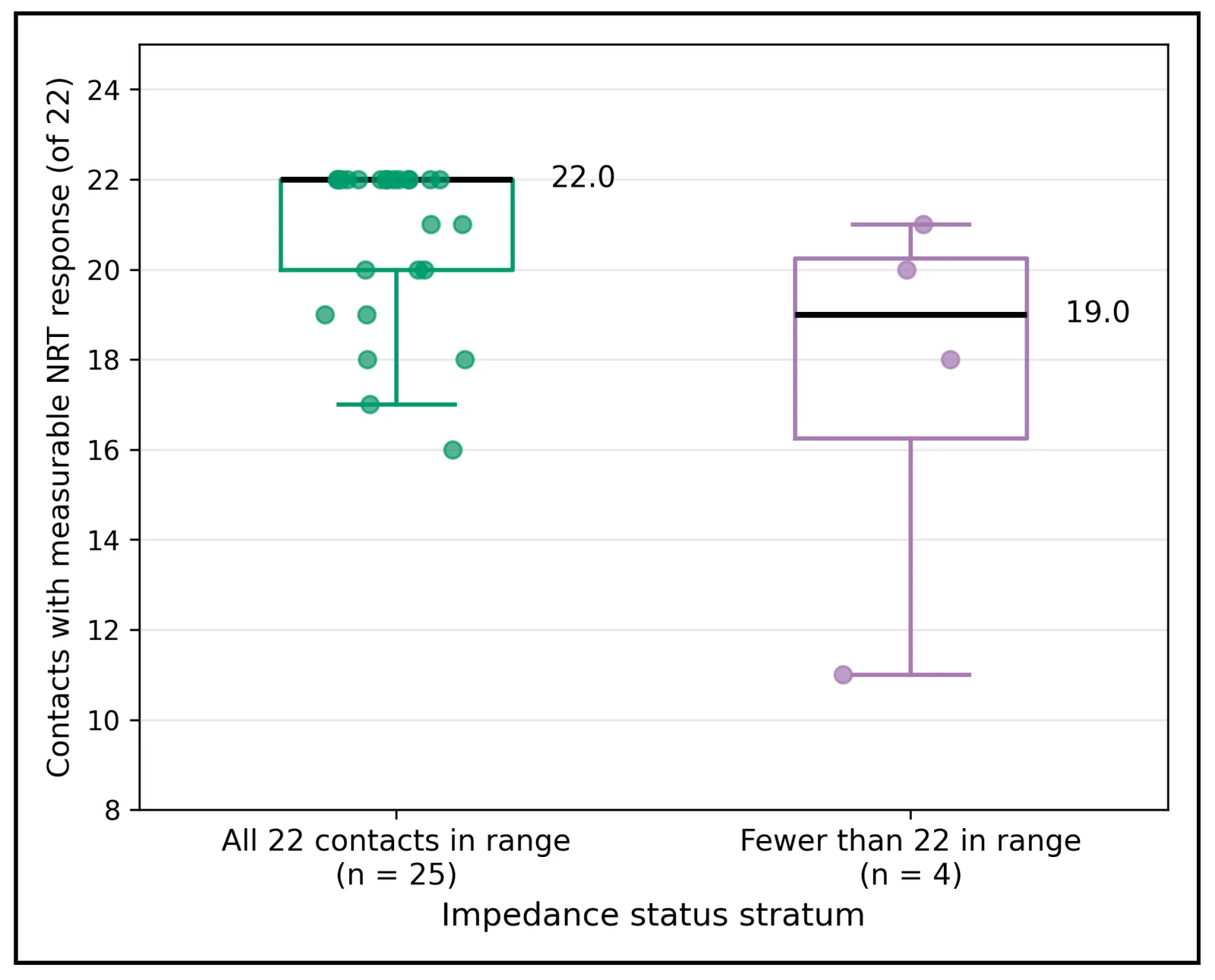
Number of contacts with a measurable NRT response (of 22), stratified by impedance status (all 22 contacts within the normal impedance range, *n* = 25, versus fewer than 22, *n* = 4). Boxes span the interquartile range, with the median as a horizontal line and whiskers extending to the most extreme values within 1.5 × IQR; every implantation is plotted as an individual point. Median values are labelled. The dichotomised comparison was borderline (U = 79.5, *p* = 0.051) and is exploratory.

In an exploratory within-array analysis, the speed–time relationship appeared to differ between arrays. Within the CI632 group the correlation was strong (ρ = −0.975, *p* = 0.005; *n* = 5), an estimate that rests on five points and should not be over-interpreted, whereas within the CI622 group it was weak and non-significant (ρ = −0.277, *p* = 0.300; *n* = 16). The impedance-stratified NRT distributions are shown in Figure 4.

All 22 contacts were within the normal impedance range in 25 of 29 implantations, and fewer than 22 (18, 19, 21, and 21) in four. Median NRT status was 22.0 responding contacts in the all-in-range stratum (IQR 20.0–22.0) and 19.0 in the sub-ceiling stratum (IQR 16.2–20.2). The dichotomised comparison was borderline and did not reach significance (U = 79.5, *p* = 0.051) although the rank-biserial effect size was −0.59; with four cases in the smaller stratum this comparison is exploratory, and part of any association is expected by construction, because a contact with out-of-range impedance is not expected to yield a measurable ECAP. The three-band age subgroup analysis is reported in Table 7.

No parameter differed significantly across the three age bands. NRT produced the largest test statistic (H = 3.31, *p* = 0.191), with medians of 20.0, 22.0 and 22.0 responding contacts across the 0–6, 6–10 and >10-year bands. Insertion time medians were 25.0, 36.5 and 30.0 s (H = 2.69, *p* = 0.261). Impedance status was 22 of 22 contacts in every band (*p* = 0.826).

### Exploratory and Sensitivity Analyses

The exploratory multivariable adjustment model for total insertion time is presented in Table 8.

The exploratory adjustment model was not significant (F-test *p* = 0.387; R^2^ = 0.217, adjusted R^2^ = 0.021) and no predictor reached significance. Cochleostomy carried the largest standardised coefficient (β = 0.56; +13.34 s), followed by insertion speed (β = −0.36; −26.29 s per additional s/electrode), CI632 (β = −0.21) and adult age (β = 0.11). Given 21 observations, four predictors and an adjusted R^2^ of 0.021, the model carries no evidential weight regarding independent determinants of insertion time; it is retained only to document that mutual adjustment did not materially alter the univariable pattern. Procedural consistency metrics are summarised in Table 9.

**Figure 5:**
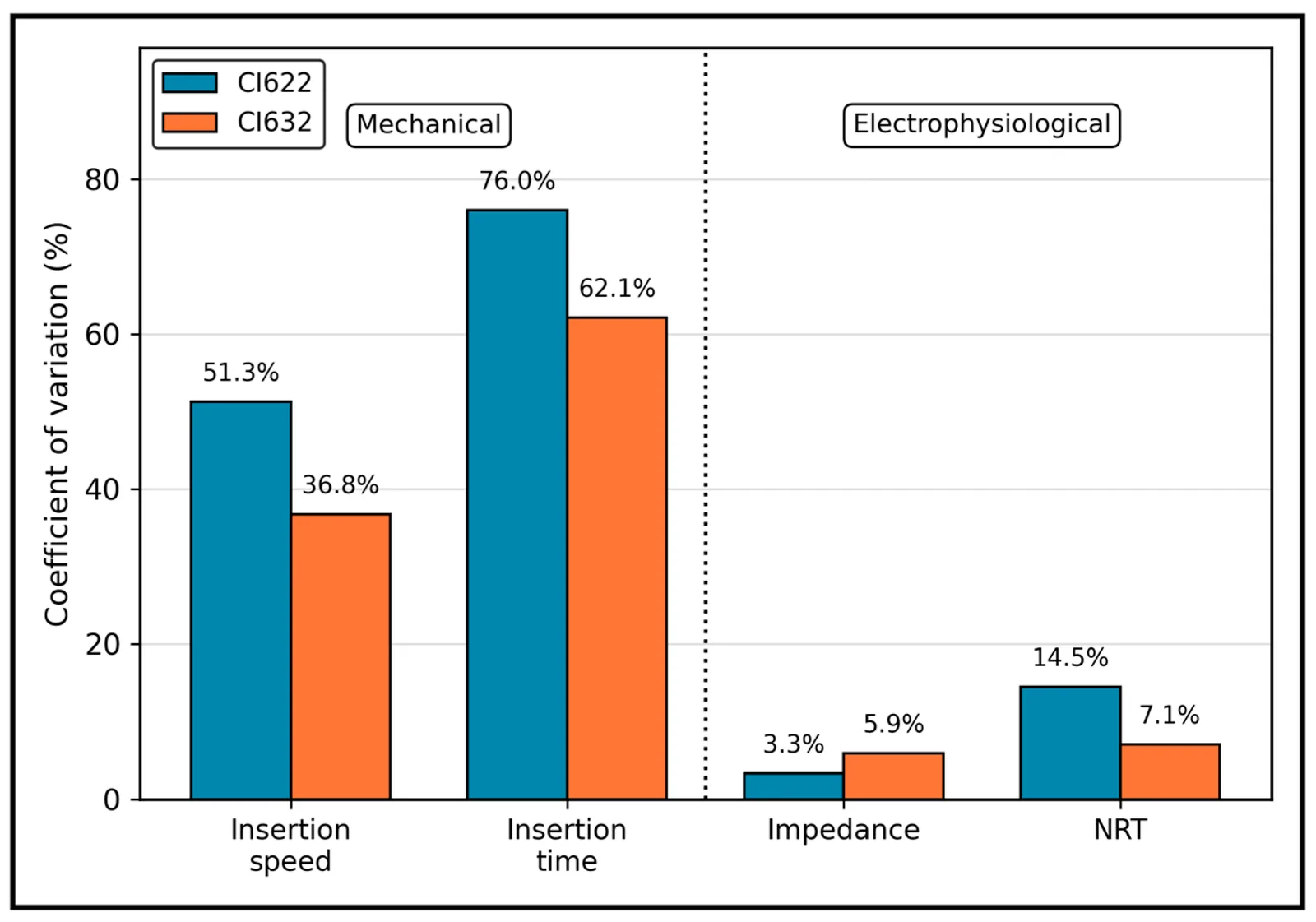
Coefficient of variation for each intraoperative parameter, shown separately for the two electrode arrays. Values above each bar give the coefficient of variation. The dotted line separates the mechanical parameters from the electrophysiological parameters.

No dispersion difference reached significance. Impedance coefficients of variation ranged from 0.0% to 5.9% and NRT from 7.1% to 14.5%, compared with 36.8% to 89.4% for the mechanical parameters. The largest test statistics were insertion speed by electrode array (W = 2.71, *p* = 0.116; CV 51.3% versus 36.8%) and insertion time by surgical approach (W = 1.67, *p* = 0.211; CV 58.8% versus 89.4%). Array-specific coefficients of variation are visualised in Figure 5.

Relative variability differed by an order of magnitude between parameter types. Mechanical parameters had coefficients of variation of 51.3% and 36.8% for insertion speed and 76.0% and 62.1% for insertion time in the CI622 and CI632 groups, respectively. Electrophysiological parameters were far more consistent, at 3.3% and 5.9% for impedance and 14.5% and 7.1% for NRT. Robustness and sensitivity analyses are summarised in Table 10.

**Figure 6:**
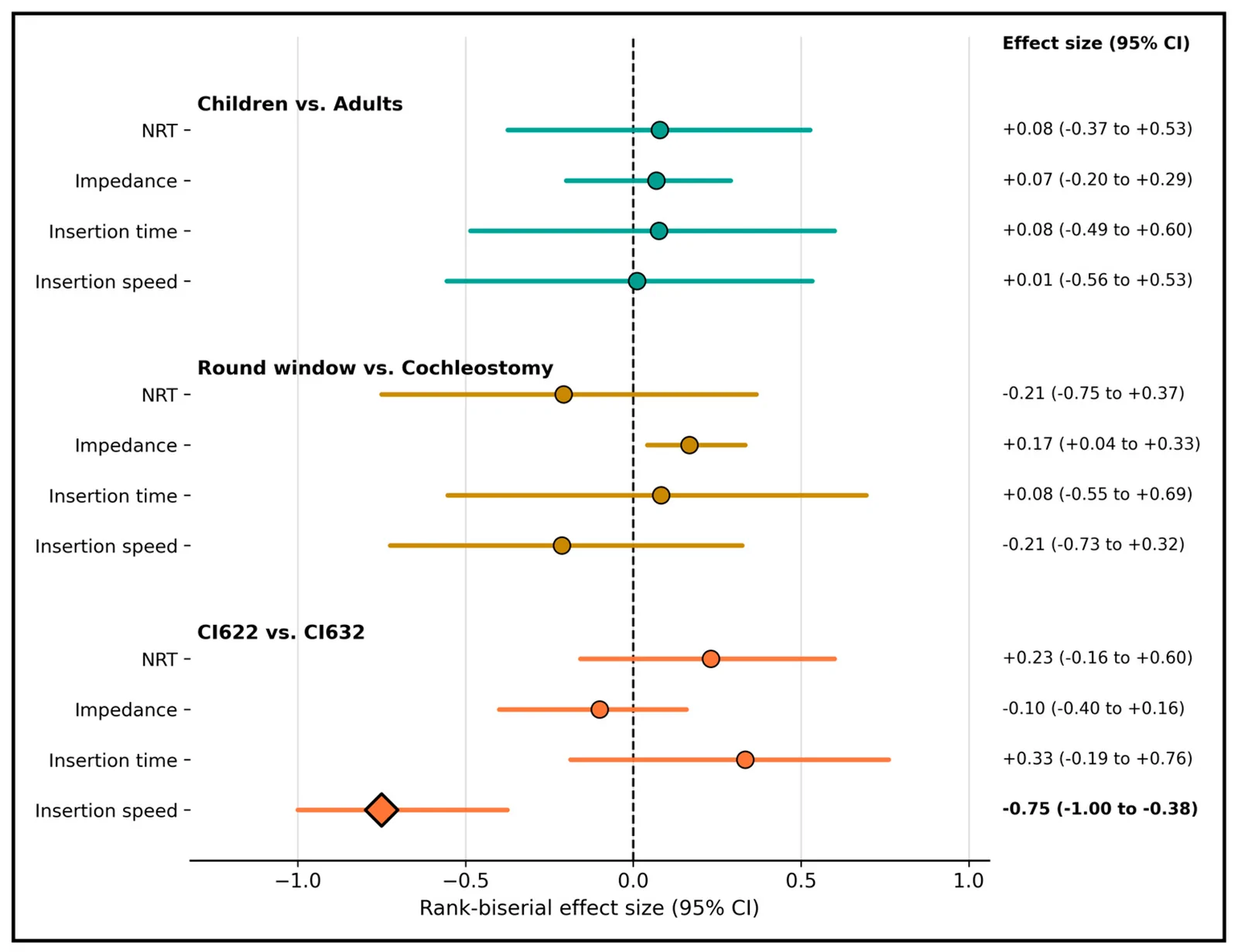
Effect size synthesis across all three group comparisons. Rank-biserial effect sizes are shown with 3000-sample bootstrap 95% confidence intervals, with numeric values given at the right. Diamonds mark comparisons whose interval excludes zero; circles mark comparisons whose interval includes zero. The dashed vertical line indicates a null effect.

The rank-biserial effect size for insertion speed was −0.750 (bootstrap 95% CI −1.000 to −0.375) and the Hodges–Lehmann difference 0.315 s/electrode (95% CI +0.15 to +0.55); both intervals excluded zero. With the outlying speed value retained, the same comparison was non-significant (*p* = 0.113). In the one-ear-per-patient analysis (*n* = 26), the speed difference persisted (U = 61.0, *p* = 0.018, r = −0.74) and the insertion time comparison remained non-significant (*p* = 0.25); all other group comparisons likewise remained non-significant. The impedance–NRT correlation was unchanged after adjustment for age band (partial ρ = 0.389 versus zero-order ρ = 0.388; *p* = 0.037) but was attenuated and no longer significant with one ear per patient (ρ = 0.371, *p* = 0.062). All group comparisons are summarised as effect sizes in Figure 6.

Of the twelve comparisons, only insertion speed between electrode arrays had a confidence interval excluding zero (−0.75, 95% CI −1.00 to −0.38). Age comparisons ranged from +0.01 to +0.08, surgical approach comparisons from −0.21 to +0.17, and the remaining electrode comparisons from −0.10 to +0.33. The approach comparisons in particular have wide intervals, reflecting the five cochleostomy cases.

## 4. Discussion

### 4.1. Analysis of Findings

The principal observation of this study is that electrode array design, rather than patient age or surgical approach, was associated with intraoperative insertion kinetics. The data suggest that the slim-modiolar CI632 was advanced roughly a third of a second faster per electrode than the slim-straight CI622; the effect size was large and its bootstrap interval excluded zero, but the comparison rests on five CI632 speed values and was no longer significant when the single excluded value was retained. It is therefore an observed association that requires confirmation rather than an established effect. Readers should therefore note that the observed array differences, including the insertion-speed effect, are sensitive to outlier handling and to the small CI632 kinetic subgroup (*n* = 5 for speed), so the estimated magnitude of the effect should be interpreted with corresponding caution. The direction of the difference is nonetheless mechanistically coherent. Slim-straight lateral wall arrays such as the CI622 are advanced along the outer cochlear wall and their progression is governed by progressive frictional contact with it [15]; the resistance encountered is further modulated by individual scala tympani dimensions, which constrain how deeply a straight array travels before contact forces rise [16]. Insertion force modelling established that array geometry and insertion technique jointly determine the resistance profile encountered during placement [17]. The CI632, by contrast, is deployed by a sheath-based controlled-release manoeuvre in which the array unfurls towards the modiolus rather than being advanced against the lateral wall, decoupling its progression from the frictional regime that governs slim-straight insertion. A shorter per-electrode dwell is therefore precisely what its design predicts. The contribution of the present data is accordingly not the direction of the difference, which the array’s design anticipates, but its quantification under routine conditions: an objective, platform-recorded estimate of the per-electrode difference (Hodges–Lehmann Δ ≈ 0.3 s/electrode), together with the observation that total insertion time did not follow the same pattern, and the exploratory suggestion that the two arrays differ in how advancement pace relates to total time. These are hypotheses for prospective testing rather than confirmed effects.

The magnitude of this difference matters because insertion speed is one of the few intraoperative variables under direct surgical control with an established relationship to cochlear trauma. Human kinematic studies have shown that surgeons cannot reliably maintain very slow, uniform advancement by hand, with involuntary micro-motions imposing a practical floor on achievable insertion speed [18]. This limitation has motivated robotic insertion systems designed to deliver consistent, controlled velocity independent of operator tremor [19], and a recent scoping review situates insertion speed alongside insertion depth as a principal determinant of both insertion forces and intracochlear pressure transients [20]. Against that background, an association between the choice of array and the speed distribution would be clinically relevant if confirmed: if slower insertion is protective, device selection may partially determine how much of that protection is achievable by hand. The present data cannot establish whether the faster CI632 deployment carries any cost or benefit, because neither intracochlear pressure, residual hearing measurement, imaging of electrode position, nor any audiological outcome was available; no claim of clinical superiority for either array is made.

A second, exploratory observation concerns the relationship between per-electrode speed and total insertion time. These quantities were only weakly related across the cohort as a whole, and the exploratory adjustment model likewise attributed little of the variance in total time to array type. Examined within each array, they appeared to behave differently: for the CI632 the relationship was strong, whereas for the CI622 it was weak and scattered. A plausible reading is that sheath-based deployment is a comparatively stereotyped manoeuvre whose duration is largely fixed by the advancement rate, whereas lateral wall insertion involves variable pauses, repositioning and tactile reassessment that decouple total time from instantaneous speed. Because the CI632 estimate rests on only five paired observations, this dissociation must be regarded as provisional and does not form part of the main findings of the study. It nonetheless offers a concrete and testable hypothesis about how the two deployment techniques differ in procedural consistency, and it aligns with the lower speed variability observed for the CI632 in the dispersion analysis.

A further exploratory observation was the modest association between impedance status and neural response telemetry, which persisted essentially unchanged after adjustment for age band but was attenuated in the one-ear-per-patient analysis and would not survive any correction for multiplicity. Impedance is increasingly regarded not merely as an integrity check but as a quantitative biomarker of the electrode–tissue interface; its subcomponents track residual hearing after implantation and distinguish access resistance from polarisation components that respond differently to intracochlear tissue reaction [21]. Longitudinal impedance behaviour has been proposed as a marker of hearing preservation on similar grounds [22], and four-point impedance measurement, which suppresses the electrode polarisation term, has been advanced as a more specific index of the intracochlear environment [23]. The concordant movement of impedance and NRT status observed here is compatible with both quantities indexing a shared underlying state of the electrode–neural interface. Two caveats apply. First, because the impedance variable is a bounded count that reached its ceiling in 25 of 29 implantations, the correlation is determined by four observations and is equivalent to a borderline two-group comparison (*p* = 0.051). Second, part of the association is expected by construction, because a contact flagged as out of range is not expected to yield a measurable ECAP. The association therefore does not, on its own, establish a combined intraoperative integrity signal; it identifies a relationship worth testing with contact-level impedance magnitudes and ECAP thresholds in a larger cohort.

Impedance also encodes spatial information, which broadens the interpretation of this coupling. Because the resistance measured at each contact depends on its local intracochlear environment, impedance profiles have been used to estimate electrode array insertion depth intraoperatively [24], and impedance-based monitoring has been evaluated as a means of tracking the progress of insertion in real time as the array advances [25]. If impedance partly reflects array position as well as tissue state, a correlation with neural response telemetry may arise both from interface integrity and from the proximity of contacts to excitable neural tissue. The present study cannot separate these contributions, since no imaging-based measure of final electrode position was available. That ambiguity is itself informative for future work: pairing intraoperative telemetry with postoperative imaging would allow the positional and interface components of the impedance–NRT relationship to be disentangled.

The study was also unable to detect a difference between surgical approaches, and this should not be read as evidence of procedural equivalence. The comparative literature remains genuinely divided. Histological examination of temporal bones has reported differing patterns of intracochlear injury between the two entry routes [26], while radiological series comparing straight arrays inserted through the round window or a cochleostomy have found broadly comparable electrode position and hearing preservation at initial activation [27]. Functional comparisons have likewise reported similar spectral resolution and speech perception between the techniques [28]. The absence of a detectable difference in the present cohort is not inconsistent with that body of work, but it must be read as underpowered rather than demonstrated: with only five cochleostomies, the confidence intervals around every approach comparison were wide, and the study had little capacity to detect any but a very large difference.

Part of the difficulty in resolving this debate is anatomical. The round window varies considerably between individuals in accessibility, orientation and the extent of overlying bony overhang, so the two approaches are not applied to comparable anatomy in practice; the choice is frequently dictated by what the surgeon encounters after posterior tympanotomy [29]. Standard technique descriptions accordingly present route selection as an individualised decision rather than a fixed protocol [30], which may partly explain why approach-related differences have been so inconsistent across published series. Finally, no age effect was detected. Neither the paediatric–adult dichotomy nor the three-band analysis revealed any difference, and the electrical parameters in particular were near-constant across every stratum. With 10 adult ears and a 6–10-year band of only two, this, too, is an absence of detectable difference rather than demonstrated invariance; within that limit, it is at least reassuring that the recorded insertion parameters in young children were not measurably worse than those in adults.

Beyond intraoperative insertion kinetics and electrophysiological stability, procedural safety remains clinically relevant and was not assessed here. Adult cochlear implantation cohorts continue to report both minor and major complications, including electrode-related events such as misplacement, migration and extrusion, alongside wound, vestibular and device-related problems [31]. Intraoperative technical advantages of one array or entry route, such as the faster per-electrode advancement recorded for the CI632 in this series, therefore need to be interpreted cautiously and within the broader context of postoperative complications and audiological outcomes, none of which were captured by the present design.

### 4.2. Study Limitations

Several limitations constrain these inferences. The sample of 29 implantations is small and the subgroups unbalanced—most consequentially five cochleostomy cases against twenty-four round window cases—so the approach comparison is underpowered and its non-significant results reflect a failure to detect differences rather than demonstrated equivalence. The single-centre design limits generalisability. Three children contributed both ears (29 ears from 26 patients), and these second-ear records were treated as independent events in the primary analysis, which could modestly inflate precision; the one-ear-per-patient sensitivity analysis left the electrode array speed difference essentially unchanged but rendered the impedance–NRT correlation non-significant. The electrode-speed difference was conditional on excluding one internally inconsistent CI632 value and rests on five CI632 speed observations, because the kinetics record was missing in four of ten CI632 sessions; although disclosed through sensitivity analysis, it is sensitive to both outlier handling and sample size, and remains replication-dependent, as does the within-array speed–time correlation resting on five paired observations. Impedance and NRT status are bounded counts that were strongly ceiling-clustered (22 of 22 in 25 and 14 implantations, respectively), compressing their variance and making rank correlations dependent on a handful of observations; contact-level impedance magnitudes and ECAP thresholds were not analysed. Finally, no postoperative audiological, imaging, trauma or complication outcome was linked to the intraoperative parameters, and 51 tests were examined without formal correction, so the two nominally significant results (*p* = 0.015 and *p* = 0.037) are consistent with chance findings and warrant hypothesis-generating interpretation only.

## 5. Conclusions

In this small single-centre series, the study was unable to detect a difference in SmartNav-recorded mechanical or electrophysiological parameters between children and adults or between round window and cochleostomy entry. With only five cochleostomies, this finding is compatible with, but does not demonstrate, procedural equivalence, and it should not be taken to show that surgical entry route has no effect on the recorded parameters.

Electrode array design was the only dimension along which a nominally significant difference emerged: the data suggest that the slim-modiolar array was advanced faster per electrode than the slim-straight array, without a corresponding difference in total insertion time or in electrophysiological status. This is a statistically supported but fragile observation, resting on five CI632 values and on the exclusion of one implausible data point, and it requires confirmation. The modest association between impedance and NRT status is an exploratory finding, consistent with chance once multiplicity is considered, and does not yet support their combined use as an intraoperative integrity signal. No clinical outcome was linked to the intraoperative parameters, so no conclusion about the clinical superiority of either array or entry route can be drawn. Prospective confirmation in a larger, balanced cohort, with one ear per patient, contact-level electrophysiological measures and linked audiological, imaging and complication outcomes, remains the necessary next step.

## Figures and Tables

**Table 1:** Baseline demographic and clinical characteristics of the cohort, overall and by age group.

Characteristic	Overall (N = 29)	Children (*n* = 19)	Adults (*n* = 10)	*p* ^1^
Male sex, *n* (%)	16 (55.2)	10 (52.6)	6 (60.0)	1.000
Female sex, *n* (%)	13 (44.8)	9 (47.4)	4 (40.0)	—
Left ear, *n* (%)	16 (55.2)	12 (63.2)	4 (40.0)	0.270
Right ear, *n* (%)	13 (44.8)	7 (36.8)	6 (60.0)	—
CI622 array, *n* (%)	19 (65.5)	13 (68.4)	6 (60.0)	0.698
CI632 array, *n* (%)	10 (34.5)	6 (31.6)	4 (40.0)	—
Round window, *n* (%)	24 (82.8)	17 (89.5)	7 (70.0)	0.306
Cochleostomy, *n* (%)	5 (17.2)	2 (10.5)	3 (30.0)	—
Age band 0–6 y, *n* (%)	13 (44.8)	13 (68.4)	—	—
Age band 6–10 y, *n* (%)	2 (6.9)	2 (10.5)	—	—
Age band > 10 y, *n* (%)	14 (48.3)	4 (21.1)	10 (100)	—

^1^ Fisher’s exact test comparing the paediatric and adult subgroups.

**Table 2:** Availability of each intraoperative parameter by electrode array (number of implantations with a recorded value; *n* (%)).

Parameter	CI622 (*n* = 19)	CI632 (*n* = 10)	All (N = 29)
Insertion angle (°)	16 (84.2)	Not computed ^1^	16 (55.2)
Insertion speed, recorded (s/el)	16 (84.2)	6 (60.0)	22 (75.9)
Insertion speed, analysed (s/el) ^2^	16 (84.2)	5 (50.0)	21 (72.4)
Insertion time (s)	16 (84.2)	6 (60.0)	22 (75.9)
Impedance status (contacts in range, of 22)	19 (100)	10 (100)	29 (100)
NRT status (responding contacts, of 22)	19 (100)	10 (100)	29 (100)

^1^ Angular insertion depth is not derived by the platform for the pre-curved CI632. ^2^ After exclusion of one implausible CI632 value (Section 2.2). Kinetics records were missing in 3 CI622 and 4 CI632 sessions (Fisher’s exact *p* = 0.193); the missingness was unrelated to deployment, insertion difficulty or any intraoperative event (Section 2.2).

**Table 3:** Intraoperative parameters compared between children and adults (Mann–Whitney U test).

Parameter	Children, Median (IQR)	Adults, Median (IQR)	U	*p*	r ^2^
Insertion angle (°) ^1^	297.0 (292.5–362.5)	324.0 (299.0–325.0)	25.0	0.821	0.09
Insertion speed (s/el)	0.49 (0.33–0.71)	0.43 (0.35–0.65)	44.5	1.000	0.01
Insertion time (s)	30.0 (21.5–37.5)	27.0 (22.5–64.5)	48.5	0.805	0.08
Impedance (contacts in range, of 22)	22.0 (22.0–22.0)	22.0 (22.0–22.0)	88.5	0.646	0.07
NRT (responding contacts, of 22)	21.0 (19.5–22.0)	22.0 (18.5–22.0)	87.5	0.733	0.08

^1^ CI622 array only (angle not computed for CI632). ^2^ Rank-biserial effect size.

**Table 4:** Intraoperative parameters were compared between surgical approaches (Mann–Whitney U test).

Parameter	Round Window, Median (IQR)	Cochleostomy, Median (IQR)	U	*p*	r
Insertion angle (°) ^1^	297.0 (292.5–341.5)	324.0 (299.0–350.0)	21.0	0.496	0.24
Insertion speed (s/el)	0.52 (0.34–0.71)	0.37 (0.32–0.49)	48.5	0.509	−0.21
Insertion time (s)	30.0 (25.0–34.0)	27.0 (20.0–55.0)	39.0	0.814	0.08
Impedance (contacts in range, of 22)	22.0 (22.0–22.0)	22.0 (22.0–22.0)	50.0	0.360	0.17
NRT (responding contacts, of 22)	21.5 (19.75–22.0)	20.0 (18.0–22.0)	72.5	0.461	−0.21

^1^ CI622 array only. Round window *n* = 24; cochleostomy *n* = 5.

**Table 5:** Intraoperative parameters compared between electrode arrays (Mann–Whitney U test).

Parameter	CI622, Median (IQR)	CI632, Median (IQR)	U	*p*	r
Insertion speed (s/el)	0.56 (0.44–0.81)	0.33 (0.17–0.34)	70.0	0.015 *	−0.75
Insertion time (s)	28.5 (19.5–35.8)	32.0 (27.8–53.5)	32.0	0.252	0.33
Impedance (contacts in range, of 22)	22.0 (22.0–22.0)	22.0 (22.0–22.0)	104.5	0.491	−0.10
NRT (responding contacts, of 22)	21.0 (18.5–22.0)	22.0 (20.3–22.0)	73.0	0.294	0.23

* Significant at α = 0.05. Angle omitted (measurable for CI622 only). CI622 *n* = 19; CI632 *n* = 10.

**Table 6:** Spearman rank-correlation matrix among intraoperative parameters, with *p*-values in parentheses.

Parameter	Angle	Speed	Time	Impedance	NRT
Angle	—				
Speed	−0.284 (0.286)	—			
Time	0.210 (0.434)	−0.381 (0.089)	—		
Impedance	−0.365 (0.165)	0.043 (0.852)	0.104 (0.644)	—	
NRT	−0.370 (0.159)	0.147 (0.526)	0.081 (0.722)	0.388 (0.037) *	—

* Significant at α = 0.05. Pairwise *n* varies (16–29) with parameter availability. *p*-values are uncorrected for multiple comparisons; no correlation remains significant at the Bonferroni-adjusted threshold (α ≈ 0.001). Impedance status is tied at the ceiling value of 22 in 25 of 29 implantations, so its correlations are determined by four observations.

**Table 7:** Subgroup analysis of intraoperative parameters across three age bands (Kruskal–Wallis test).

Parameter	0–6 y, Median (*n*)	6–10 y, Median (*n*)	>10 y, Median (*n*)	H	*p*
Insertion angle (°) ^1^	297.0 (5)	325.5 (2)	324.0 (9)	0.93	0.628
Insertion speed (s/el)	0.39 (9)	0.56 (2)	0.49 (10)	0.30	0.861
Insertion time (s)	25.0 (9)	36.5 (2)	30.0 (11)	2.69	0.261
Impedance (contacts in range, of 22)	22.0 (13)	22.0 (2)	22.0 (14)	0.38	0.826
NRT (responding contacts, of 22)	20.0 (13)	22.0 (2)	22.0 (14)	3.31	0.191

^1^ CI622 array only. The 6–10 y band contains only two recipients; estimates for that band are unstable.

**Table 8:** Exploratory multivariable linear regression (adjustment analysis): predictors of total insertion time (seconds).

Predictor	β (95% CI)	Std. β	t	*p*
Intercept	46.96 (13.79 to 80.13)	—	3.00	0.009
Adult (versus child)	2.55 (−23.75 to 28.86)	0.11	0.21	0.840
Cochleostomy (versus RW)	13.34 (−17.39 to 44.07)	0.56	0.92	0.371
CI632 (versus CI622)	−4.91 (−37.29 to 27.48)	−0.21	−0.32	0.752
Insertion speed (s/el)	−26.29 (−67.26 to 14.68)	−0.36	−1.36	0.193

Model: *n* = 21; R^2^ = 0.217; adjusted R^2^ = 0.021; overall F-test *p* = 0.387. RW, round window.

**Table 9:** Procedural consistency analysis: coefficient of variation and equality of dispersion (Brown–Forsythe test).

Parameter and Stratum	CV Group 1	CV Group 2	Levene W	*p*
**By approach (RW versus CS)**				
Insertion speed	60.6%	56.1%	0.62	0.440
Insertion time	58.8%	89.4%	1.67	0.211
Impedance	4.7%	0.0%	0.67	0.422
NRT	12.4%	13.3%	0.13	0.723
**By electrode (CI622 versus CI632)**				
Insertion speed	51.3%	36.8%	2.71	0.116
Insertion time	76.0%	62.1%	0.07	0.795
Impedance	3.3%	5.9%	0.63	0.436
NRT	14.5%	7.1%	1.50	0.231

Group 1 = round window (RW) or CI622; group 2 = cochleostomy (CS) or CI632. CV, coefficient of variation.

**Table 10:** Robustness of the electrode design and impedance–NRT findings: bootstrap effect sizes, Hodges–Lehmann estimates, sensitivity analyses (outlier retained; one ear per patient), and partial correlation.

Analysis	Estimate	95% CI	*p*
Rank-biserial r, speed, CI622 versus CI632	−0.750	−1.000 to −0.375 ^1^	0.015
Hodges–Lehmann Δ speed (s/electrode)	+0.315	+0.15 to +0.55 ^1^	—
Sensitivity: Mann–Whitney with outlier retained	U = 70.0	—	0.113
Sensitivity: speed, one ear per patient (CI622 *n* = 14 versus CI632 *n* = 5)	U = 61.0; r = −0.74	—	0.018
Sensitivity: time, one ear per patient (CI622 *n* = 14 versus CI632 *n* = 6)	U = 28.0; r = +0.33	—	0.25
Impedance–NRT, zero-order Spearman ρ	+0.388	—	0.037
Impedance–NRT, partial ρ given age band	+0.389	—	0.037
Impedance–NRT, Spearman ρ, one ear per patient (*n* = 26)	+0.371	—	0.062

^1^ 2000-sample bootstrap percentile interval. Δ = CI622 minus CI632; positive values denote slower per-electrode advancement for CI622. One ear per patient: the first-implanted ear was retained for each of the three bilaterally implanted children. The outlier-retained analysis adds the excluded CI632 value (4.5 s/electrode) to the CI632 group (*n* = 6).

## Data Availability

The data presented in this study are available on request from the corresponding author. The data are not publicly available due to privacy restrictions.

## References

[1] World Health Organization (2025). Deafness and Hearing Loss.

[2] Buchman C.A., Gifford R.H., Haynes D.S., Lenarz T., O’Donoghue G., Adunka O., Biever A., Briggs R.J., Carlson M.L., Dai P. (2020). Unilateral Cochlear Implants for Severe, Profound, or Moderate Sloping to Profound Bilateral Sensorineural Hearing Loss: A Systematic Review and Consensus Statements. JAMA Otolaryngol. Head Neck Surg..

[3] Tang D., Tran Y., Lo C., Wang J., McMahon C., Gopinath B. (2024). The Benefits of Cochlear Implantation for Adults: A Systematic Umbrella Review. Ear Hear..

[4] Rask-Andersen H., Liu W., Erixon E., Kinnefors A., Pfaller K., Schrott-Fischer A., Glueckert R. (2012). Human Cochlea: Anatomical Characteristics and Their Relevance for Cochlear Implantation. Anat. Rec..

[5] Adunka O., Unkelbach M.H., Mack M., Hambek M., Gstoettner W., Kiefer J. (2004). Cochlear Implantation via the Round Window Membrane Minimizes Trauma to Cochlear Structures: A Histologically Controlled Insertion Study. Acta Otolaryngol..

[6] Kontorinis G., Lenarz T., Stöver T., Paasche G. (2011). Impact of the Insertion Speed of Cochlear Implant Electrodes on the Insertion Forces. Otol. Neurotol..

[7] Rajan G.P., Kontorinis G., Kuthubutheen J. (2013). The Effects of Insertion Speed on Inner Ear Function during Cochlear Implantation: A Comparison Study. Audiol. Neurotol..

[8] Banakis Hartl R.M., Greene N.T. (2022). Measurement and Mitigation of Intracochlear Pressure Transients during Cochlear Implant Electrode Insertion. Otol. Neurotol..

[9] Concheri S., Brotto D., Ariano M., Daloiso A., Di Pasquale Fiasca V.M., Sorrentino F., Coppadoro B., Trevisi P., Zanoletti E., Franchella S. (2024). Intraoperative Measurement of Insertion Speed in Cochlear Implant Surgery: A Preliminary Experience with Cochlear SmartNav. Audiol. Res..

[10] Zawawi F., Bukhari A.F., Khairy S.A., Garrada M. (2025). Surgeon-Performed Intraoperative Cochlear Implant Testing Using SmartNav: A Step toward Streamlining Surgical Workflow. Int. J. Pediatr. Otorhinolaryngol..

[11] Wanna G.B., Noble J.H., Carlson M.L., Gifford R.H., Dietrich M.S., Haynes D.S., Dawant B.M., Labadie R.F. (2014). Impact of Electrode Design and Surgical Approach on Scalar Location and Cochlear Implant Outcomes. Laryngoscope.

[12] Jiam N.T., Jiradejvong P., Pearl M.S., Limb C.J. (2016). The Effect of Round Window vs Cochleostomy Surgical Approaches on Cochlear Implant Electrode Position: A Flat-Panel Computed Tomography Study. JAMA Otolaryngol. Head Neck Surg..

[13] Hodges J.L., Lehmann E.L. (1963). Estimates of Location Based on Rank Tests. Ann. Math. Stat..

[14] Wendt H.W. (1972). Dealing with a Common Problem in Social Science: A Simplified Rank-Biserial Coefficient of Correlation Based on the U Statistic. Eur. J. Soc. Psychol..

[15] Skarzynski H., Lorens A., Matusiak M., Porowski M., Skarzynski P.H., James C.J. (2014). Cochlear Implantation with the Nucleus Slim Straight Electrode in Subjects with Residual Low-Frequency Hearing. Ear Hear..

[16] Morrel W.G., Holder J.T., Dawant B.M., Noble J.H., Labadie R.F. (2020). Effect of Scala Tympani Height on Insertion Depth of Straight Cochlear Implant Electrodes. Otolaryngol. Head Neck Surg..

[17] Roland J.T. (2005). A Model for Cochlear Implant Electrode Insertion and Force Evaluation: Results with a New Electrode Design and Insertion Technique. Laryngoscope.

[18] Kesler K., Dillon N.P., Fichera L., Labadie R.F. (2017). Human Kinematics of Cochlear Implant Surgery: An Investigation of Insertion Micro-Motions and Speed Limitations. Otolaryngol. Head Neck Surg..

[19] Kashani R.G., Henslee A., Nelson R.F., Hansen M.R. (2024). Robotic Assistance during Cochlear Implantation: The Rationale for Consistent, Controlled Speed of Electrode Array Insertion. Front. Neurol..

[20] Hrncirik F., Nagy L., Grimes H.L., Iftikhar H., Muzaffar J., Bance M. (2024). Impact of Insertion Speed, Depth, and Robotic Assistance on Cochlear Implant Insertion Forces and Intracochlear Pressure: A Scoping Review. Sensors.

[21] Schraivogel S., Aebischer P., Weder S., Caversaccio M., Wimmer W. (2023). Cochlear Implant Electrode Impedance Subcomponents as Biomarker for Residual Hearing. Front. Neurol..

[22] Wimmer W., Sclabas L., Caversaccio M., Weder S. (2022). Cochlear Implant Electrode Impedance as Potential Biomarker for Residual Hearing. Front. Neurol..

[23] Tan E., Bester C., Collins A., Razmovski T., O’Leary S. (2024). Four-Point Impedance: A Potential Biomarker for Residual Hearing after Cochlear Implantation. Otol. Neurotol..

[24] Aebischer P., Meyer S., Caversaccio M., Wimmer W. (2021). Intraoperative Impedance-Based Estimation of Cochlear Implant Electrode Array Insertion Depth. IEEE Trans. Biomed. Eng..

[25] Gottfried T.M., Galeazzi P., Föger A., Dejaco D., Rasse T., Fischer N., Schmutzhard J. (2024). Evaluation of an Impedance-Based Method to Monitor the Insertion of the Electrode Array during Cochlear Implantation. Eur. Arch. Otorhinolaryngol..

[26] Richard C., Fayad J.N., Doherty J., Linthicum F.H. (2012). Round Window versus Cochleostomy Technique in Cochlear Implantation: Histologic Findings. Otol. Neurotol..

[27] Hassepass F., Aschendorff A., Bulla S., Arndt S., Maier W., Laszig R., Beck R. (2015). Radiologic Results and Hearing Preservation with a Straight Narrow Electrode via Round Window versus Cochleostomy Approach at Initial Activation. Otol. Neurotol..

[28] Demir B., Yüksel M., Atilgan A., Ciprut A., Batman C. (2021). Spectral Resolution and Speech Perception after Cochlear Implantation Using the Round Window versus Cochleostomy Technique. J. Laryngol. Otol..

[29] Luers J.C., Hüttenbrink K.B., Beutner D. (2018). Surgical Anatomy of the Round Window—Implications for Cochlear Implantation. Clin. Otolaryngol..

[30] Mangus B., Rivas A., Tsai B.S., Haynes D.S., Roland J.T. (2012). Surgical Techniques in Cochlear Implants. Otolaryngol. Clin. N. Am..

[31] Dalgic A., Basaran Bozkurt E., Aliyeva A., Görgülü E., Ceylan M.E., Edizer D.T., Olgun L. (2023). Complications after Cochlear Implantation in Adult Patients: A Retrospective Study. J. Laryngol. Otol..

